# Supplementary material for: Altered frontolimbic activity during virtual reality-based contextual fear learning in patients with posttraumatic stress disorder
Source: Psychol Med. 2023 Jan 5;53(13):6345–55. doi: 10.1017/S0033291722003695 (PMC10520602; doi:10.1017/S0033291722003695)
Supplement: Supplementary file 1 [file S0033291722003695sup.zip › S0033291722003695sup003.docx]

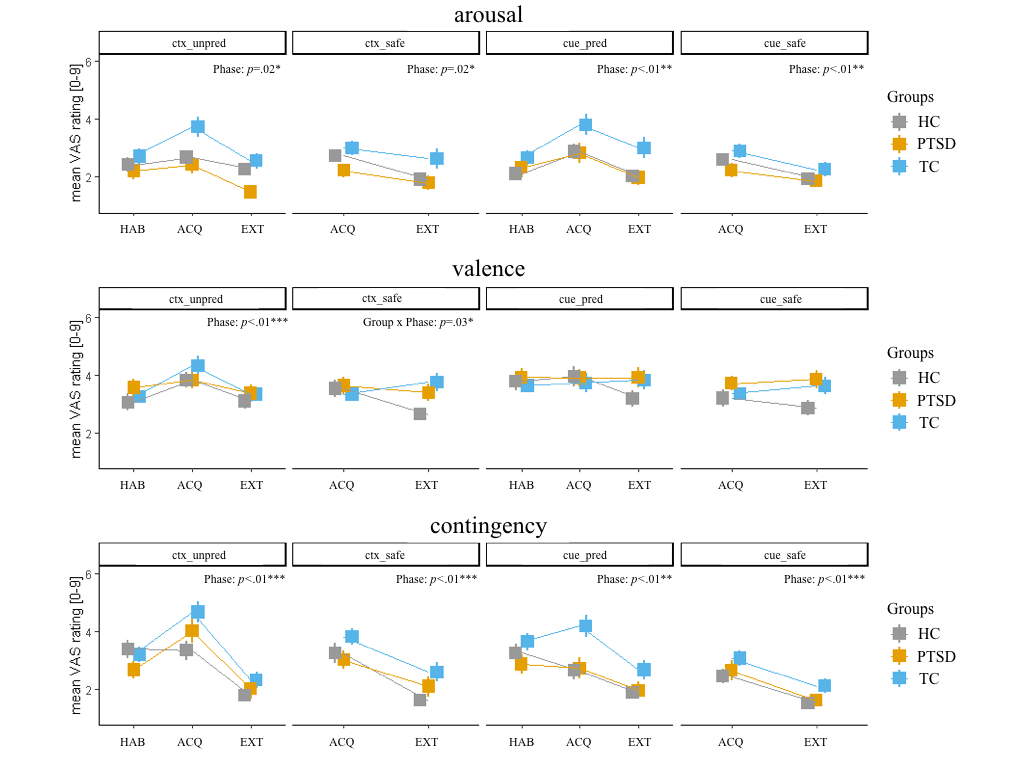


**Suppl. Figure 2a.** Arousal, valence and contingency ratings across each of the four conditions (ctx_unpred, ctx_safe, cue_pred, cue_safe), each of the three phases (HAB, ACQ, EXT) and each group (HC, PTSD, TC).

[**Abbreviations:** ACQ – Acquisition; CTX – Context; EXT – Extinction; HAB – Habituation; HC – Healthy control subjects without trauma experience; p_GG_ – Greenhouse-Geisser correction; pred – Predictable; PTSD – patients with PTSD; SCR – Skin conductance response; TC – healthy control subjects with trauma experience; unpred – Unpredictable; VAS – Visual Analogue Scale]


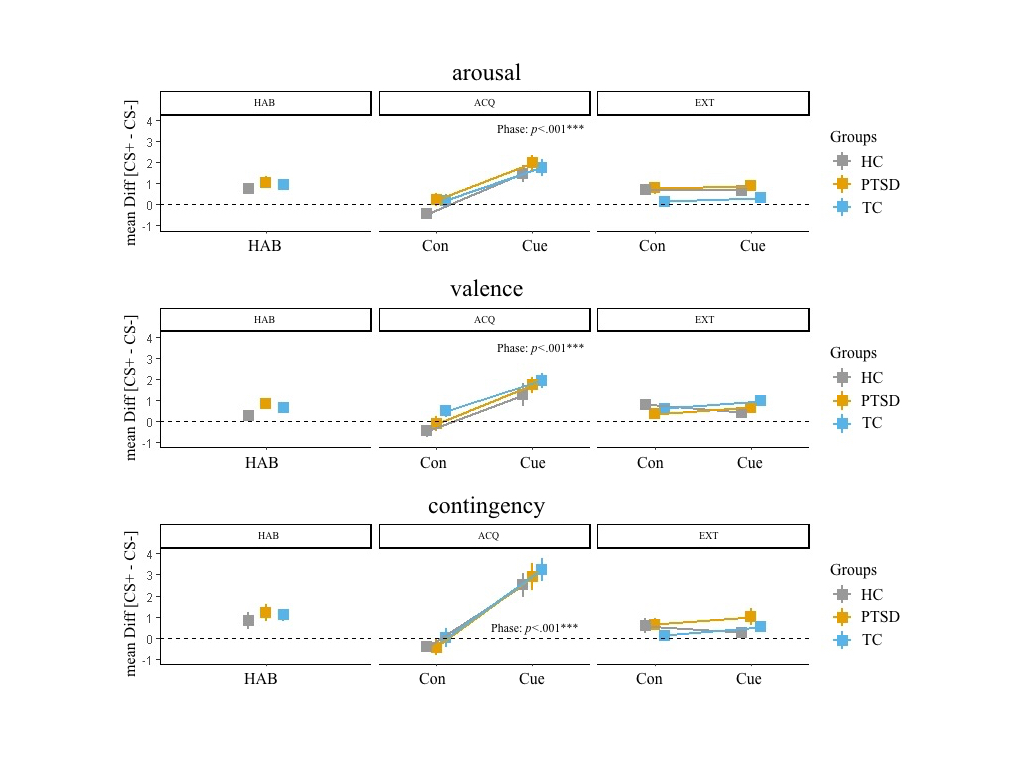


**Suppl. Figure 2b.** Difference scores (CS+ - CS-) for arousal, valence and contingency ratings across each of the three phases (HAB, ACQ, EXT) and each group (HC, PTSD, TC).

[**Abbreviations:** ACQ – Acquisition; Con – Context; EXT – Extinction; HAB – Habituation; HC – Healthy control subjects without trauma experience; PTSD – patients with PTSD; TC – healthy control subjects with trauma experience]
