# Supplementary material for: Altered frontolimbic activity during virtual reality-based contextual fear learning in patients with posttraumatic stress disorder
Source: Psychol Med. 2023 Jan 5;53(13):6345–55. doi: 10.1017/S0033291722003695 (PMC10520602; doi:10.1017/S0033291722003695)
Supplement: Supplementary file 1 [file S0033291722003695sup.zip › S0033291722003695sup005.docx]

|  | | **Groups** | | | | | | | | | | | | |  | | | | | | | | | |  |
| --- | --- | --- | --- | --- | --- | --- | --- | --- | --- | --- | --- | --- | --- | --- | --- | --- | --- | --- | --- | --- | --- | --- | --- | --- | --- |
|  | | **PTSD**  [N=20] | | | | **TC**  [N=21] | | | | **HC**  [N=22] | | | | **Analyses** | | | | | | | | | | |  |
|  |  | *M* | *SD* | *n* | *(%)* | *M* | *SD* | *n* | *(%)* | *M* | *SD* | *n* | *(%)* | *X^2^* | | *F* | *T* | *Df* | *p* | *Cont.* | *Diff,* | *CI [-95%; +95%]* | *p_Tukey HSD_* | *Hedges’ g* |  |
| NEO-FFI - Neuroticism |  | 26.6 | 9.2 | 18 |  | 19.0 | 9.4 | 21 |  | 12.8 | 6.2 | 20 |  |  | | 12.87 |  | 2 | <.001 | T-H | 6.3 | -0.0; 12.6 | .051 | 0.77 |  |
|  | | | | | | | | | | | | | | | | | | | | P-H | 13.9 | 7.3; 20.4 | <.001 | 1.77 |  |
|  |  |  |  |  |  |  |  |  |  |  |  |  |  |  |  |  |  |  |  | P-T | 7.6 | 1.1; 14.1 | .019 | 0.89 |  |
| NEO-FFI - Extraversion |  | 22.9 | 8.2 | 16 |  | 26.7 | 7.2 | 21 |  | 29.1 | 7.4 | 21 |  |  | | 3.03 |  | 2 | .056 |  |  |  |  |  |  |
| NEO-FFI - Openness to experience |  | 27.9 | 9.3 | 17 |  | 31.2 | 7.5 | 21 |  | 29.5 | 7.9 | 22 |  |  | | 0.77 |  | 2 | .47 |  |  |  |  |  |  |
| NEO-FFI - Agreeableness |  | 31.4 | 7.1 | 17 |  | 32.5 | 6.4 | 20 |  | 37.5 | 6.3 | 22 |  |  | | 4.98 |  | 2 | .010 | T-H | -5.0 | -9.8; -0.1 | .045 | 0.79 |  |
|  | | | | | | | | | | | | | | | | | | | | P-H | -6.1 | -11.2;-1.0 | .014 | 0.92 |  |
|  |  |  |  |  |  |  |  |  |  |  |  |  |  |  |  |  |  |  |  | P-T | -1.2 | -6.4; 4.1 | .86 | 0.16 |  |
| NEO-FFI - Conscientiousness |  | 30.6 | 8.0 | 18 |  | 32.6 | 5.3 | 21 |  | 34.8 | 5.8 | 22 |  |  | | 2.23 |  | 2 | .12 |  |  |  |  |  |  |
| **Neuropsychological Assessments** | | | | | | | | | | | | | | | | | | | | | | | | |  |
| PRM | Mean correct latency | 2.45 | 0.74 | 18 |  | 2.11 | 0.59 | 21 |  | 2.19 | 0.53 | 21 |  |  | | 1.60 |  | 2 | .21 | | | | | |  |
|  | Percent correct | 90.3 | 10.4 | 18 |  | 94.8 | 4.15 | 21 |  | 94.0 | 9.18 | 21 |  | 12.64 | |  |  | 8 | .13 | | | | | |  |
| PRM delayed | Mean correct latency | 2.13 | 0.49 | 18 |  | 2.02 | 0.55 | 21 |  | 2.24 | 1.27 | 21 |  |  | | 0.36 |  | 2 | .70 | | | | | |  |
|  | Percent correct | 75.5 | 16.0 | 18 |  | 85.2 | 13.1 | 21 |  | 82.9 | 14.8 | 21 |  | 13.66 | |  |  | 16 | .62 | | | | | |  |
| SSP | Span length | 5.61 | 1.85 | 18 |  | 6.57 | 1.21 | 21 |  | 6.63 | 1.34 | 19 |  |  | | 2.79 |  | 2 | .07 | | | | | |  |
|  | Total errors | 12.8 | 5.87 | 18 |  | 15.1 | 7.11 | 21 |  | 13.8 | 5.64 | 19 |  |  | | 0.67 |  | 2 | .52 | | | | | |  |
|  | Mean time to first response | 2.42 | 0.66 | 18 |  | 2.73 | 0.40 | 21 |  | 2.90 | 0.69 | 19 |  |  | | 3.07 |  | 2 | .055 | | | | | |  |
|  | Mean time to last response | 3.14 | 1.03 | 18 |  | 3.50 | 0.63 | 21 |  | 3.58 | 0.73 | 19 |  |  | | 1.57 |  | 2 | .22 | | | | | |  |
|  | Total usage errors | 2.72 | 2.11 | 18 |  | 2.24 | 1.34 | 21 |  | 2.53 | 1.95 | 19 |  |  | | 0.36 |  | 2 | 0.70 | | | | | |  |
| SRM | Mean correct latency | 2.00 | 0.44 | 18 |  | 2.09 | 0.58 | 21 |  | 2.06 | 0.51 | 21 |  |  | | 0.17 |  | 2 | .84 | | | | | |  |
|  | Percent correct | 78.3 | 10.6 | 18 |  | 79.3 | 8.70 | 21 |  | 76.7 | 9.79 | 21 |  | 17.09 | |  |  | 18 | .52 | | | | | |  |
| PAL | First trial memory score | 19.0 | 2.68 | 18 |  | 20.6 | 3.75 | 21 |  | 20.6 | 5.31 | 21 |  |  | | 0.95 |  | 2 | .39 | | | | | |  |
|  | Mean trials to success | 1.62 | 0.44 | 18 |  | 1.46 | 0.32 | 21 |  | 1.48 | 0.62 | 21 |  |  | | 0.62 |  | 2 | .54 | | | | | |  |
|  | Total error (adjusted) | 15.6 | 12.6 | 18 |  | 9.76 | 8.50 | 21 |  | 12.0 | 16.3 | 21 |  |  | | 1.01 |  | 2 | .37 | | | | | |  |
|  | Total trials | 12.8 | 3.26 | 18 |  | 11.7 | 2.59 | 21 |  | 11.6 | 4.25 | 21 |  |  | | 0.68 |  | 2 | .51 | | | | | |  |

**Suppl. Table 1.** Assessment of personality traits and Neuropsychological assessments.

**[Abbreviations:** Cont. – Contrast; HC – Healthy control subjects; NEO-FFI – Neuroticism-Extraversion-Openness to experience Five-Factor Inventory; PAL – Paired Associates Learning; PRM – Pattern Recognition Memory; PTSD – patients with PTSD; SRM – Spatial Recognition Memory; SSP – Spatial Span; TC – Trauma control subjects]
