# Supplementary material for: Altered frontolimbic activity during virtual reality-based contextual fear learning in patients with posttraumatic stress disorder
Source: Psychol Med. 2023 Jan 5;53(13):6345–55. doi: 10.1017/S0033291722003695 (PMC10520602; doi:10.1017/S0033291722003695)
Supplement: Supplementary file 1 [file S0033291722003695sup.zip › S0033291722003695sup006.docx]

| **Debriefing** | | **PTSD** | | | | **TC** | | | | **HC** | | | | **Analysis** | | | |
| --- | --- | --- | --- | --- | --- | --- | --- | --- | --- | --- | --- | --- | --- | --- | --- | --- | --- |
|  |  | ***M*** | ***SD*** | ***n*** | ***%*** | ***M*** | ***SD*** | ***n*** | ***%*** | ***M*** | ***SD*** | ***n*** | ***%*** | ***X^2^*** | ***F*** | ***Df*** | ***p*** |
| 1. How many different architects designed the rooms? |  | 6.04 | 2.37 | 14 |  | 6.69 | 1.76 | 18 |  | 5.25 | 1.77 | 20 |  |  | 2.62 | 2 | .08 |
| 2. How quickly did you manage to distinguish the rooms from each other? | During Context ACQ |  |  | 10 | 52.6 |  |  | 14 | 70.0 |  |  | 17 | 77.3 | 6.07 |  | 4 | .19 |
|  | During Cue ACQ |  |  | 7 | 36.9 |  |  | 6 | 30.0 |  |  | 5 | 22.7 |  |  |  |  |
|  | Not at all |  |  | 2 | 10.5 |  |  | 0 | 0.0 |  |  | 0 | 0.0 |  |  |  |  |
| 3. Did you find the instructions understandable?  [1 “difficult”; 10 “easy”] |  | 9.00 | 1.34 | 20 |  | 9.7 | 0.80 | 20 |  | 9.24 | 1.22 | 21 |  |  | 0.53 | 1 | .39 |
| 4. Did you find the ratings understandable?  [1 “difficult”; 10 “easy”] |  | 8.15 | 1.87 | 20 |  | 8.7 | 1.72 | 20 |  | 8.95 | 1.17 | 22 |  |  | 2.65 | 1 | .11 |
| 5. How well did you get along with the keyboard?  [1 “very badly”; 10 “very good”] |  | 8.80 | 1.61 | 20 |  | 8.35 | 2.32 | 20 |  | 9.45 | 1.06 | 22 |  |  | 1.58 | 1 | .21 |
| 6. How exhausting did you find the experiment?  [1 “very exhausting”; 10 “not exhausting at all”] |  | 5.00 | 2.81 | 19 |  | 6.40 | 2.04 | 20 |  | 6.14 | 2.10 | 21 |  |  | 2.26 | 1 | .14 |
| 7. How attentive were you during the experiment?  [1 “not at all”; 10 “very”] |  | 7.21 | 2.15 | 19 |  | 7.65 | 1.53 | 20 |  | 7.36 | 1.33 | 22 |  |  | 0.07 | 1 | .80 |

**Supplementary Table 2.** Results of debriefing questionnaire asked at the end of the habituation (Question 1) and at the end of acquisition (Question 2-7).

[Abbreviations: HC – Healthy control subjects without trauma experience; PTSD – patients with PTSD; TC – healthy control subjects with trauma experience]
