# Supplementary material for: Altered frontolimbic activity during virtual reality-based contextual fear learning in patients with posttraumatic stress disorder
Source: Psychol Med. 2023 Jan 5;53(13):6345–55. doi: 10.1017/S0033291722003695 (PMC10520602; doi:10.1017/S0033291722003695)
Supplement: Supplementary file 1 [file S0033291722003695sup.zip › S0033291722003695sup007.docx]

| \| **US** \| \| \| \| \| \| \| \| --- \| --- \| --- \| --- \| --- \| --- \| --- \| \|  \| **Groups** \|  \| **HAB** \| **ACQ Con** \| **ACQ Cue** \| **Analyses** \| \|  \| **n** \| **M (SD)** \| **M (SD)** \| **M (SD)** \|  \| \| **Intensity (in mA)** \|  \|  \|  \|  \|  \| \| PTSD \| [n=19] \| 4.68 (3.39) \| - \| - \| F(2, 58)= 0.54, p=.59 \| \| TC \| [n=20] \| 4.80 (2.41) \| - \| - \| \| HC \| [n=22] \| 3.96 (2.72) \| - \| - \| \| **Pain** \| \| \| \| \| \| \| PTSD \| [n=17] \| 7.29 (0.77) \| 5.47 (1.94) \| 5.94 (1.71) \| Group: F(2, 54)= 2.29, p=.11  **Phase: F(2, 108)= 31.27, p<.001*****  HAB > ACQ_Con_ + ACQ_Cue_  **GroupxPhase: F(4, 108)= 3.05, p=.02***  TC_ACQ_con_ > PTSD_ACQ_con_ + HC_ACQ_con_  TC_ACQ_cue_ > PTSD_ACQ_cue_ + HC_ACQ_cue_ \| \| TC \| [n=20] \| 7.10 (0.45) \| 6.55 (0.76) \| 6.55 (0.76) \| \| HC \| [n=20] \| 7.25 (0.44) \| 5.40 (2.06) \| 5.50 (1.82) \| \| **Valence** \| \| \| \| \| \| \| PTSD \| [n=17] \| 7.29 (0.69) \| 5.76 (1.92) \| 6.18 (1.88) \| Group: F(2, 54)= 3.02, p=.057  **Phase: F(2, 108)= 18.46, p_GG_<.001*****  HAB > ACQ_Con_ + ACQ_Cue_  **GroupxPhase: F(4, 108)= 3.06, p_GG_=.031***  TC_ACQ_con_ > PTSD_ACQ_con_ + HC_ACQ_con_  TC_ACQ_cue_ > HC_ACQ_cue_ \| \| TC \| [n=20] \| 7.10 (0.45) \| 6.75 (1.02) \| 6.85 (1.18) \| \| HC \| [n=20] \| 7.10 (0.55) \| 5.60 (1.90) \| 5.35 (2.21) \|   **Suppl. Table 3a.** Intensity (in Milliampere), pain intensity ratings and valence ratings of the US during HAB and ACQ.  [**Abbreviations:** ACQ – Acquisition; Con – Context; EXT – Extinction; HAB – Habituation; HC – Healthy control subjects without trauma experience; mA – Milliampere; PTSD – patients with PTSD; TC – healthy control subjects with trauma experience; US – Unconditioned Stimulus]  **Arousal** | | | | | | |
| --- | --- | --- | --- | --- | --- | --- | --- | --- | --- | --- | --- | --- | --- | --- | --- | --- | --- | --- | --- | --- | --- | --- | --- | --- | --- | --- | --- | --- | --- | --- | --- | --- | --- | --- | --- | --- | --- | --- | --- | --- | --- | --- | --- | --- | --- | --- | --- | --- | --- | --- | --- | --- | --- | --- | --- | --- | --- | --- | --- | --- | --- | --- | --- | --- | --- | --- | --- | --- | --- | --- | --- | --- | --- | --- | --- | --- | --- | --- | --- | --- | --- | --- | --- | --- | --- | --- | --- | --- | --- | --- | --- | --- |
|  | **Groups** |  | **HAB** | **ACQ** | **EXT** | **Analyses** |
|  |  | **n** | **M (SD)** | **M (SD)** | **M (SD)** |  |
|  | **CTX_unpred** |  |  |  |  |  |
|  | PTSD | [n=14] | 2.21 (1.73) | 2.46 (1.68) | 1.54 (0.91) | Group: F(2, 41)= 1.40, p=.26  **Phase: F(2, 82)= 4.33, p_GG_=.022***  ACQ > HAB + EXT  GroupxPhase: F(4, 82)= 1.38, p=.25 |
|  | TC | [n=17] | 2.41 (1.24) | 3.47 (2.22) | 2.24 (1.44) |  |
|  | HC | [n=13] | 2.15 (1.25) | 2.27 (0.90) | 2.23 (1.13) |  |
|  | **CTX_safe** | | | | | |
|  | PTSD | [n=16] | - | 2.31 (1.52) | 1.69 (1.40) | Group: F(2, 46)= 0.80, p=.46  **Phase: F(1, 46)= 6.45, p=.015***  ACQ > EXT  GroupxPhase: F(2, 46)= 0.33, p=.72 |
|  | TC | [n=18] | - | 2.72 (1.22) | 2.42 (2.10) |  |
|  | HC | [n=15] | - | 2.60 (1.45) | 1.90 (1.14) |  |
|  | **CUE_pred** | | | | | |
|  | PTSD | [n=14] | 2.46 (1.83) | 2.83 (1.83) | 2.00 (1.40) | Group: F(2, 42)= 1.30, p=.28  **Phase: F(2, 82)= 6.03, p=.004****  ACQ > HAB + EXT  GroupxPhase: F(4, 82)= 0.56, p=.69 |
|  | TC | [n=17] | 2.50 (1.35) | 3.62 (2.33) | 2.79 (2.27) |  |
|  | HC | [n=13] | 1.85 (0.90) | 2.62 (1.34) | 2.04 (1.25) |  |
|  | **CUE_safe** | | | | | |
|  | PTSD | [n=16] | - | 2.28 (1.48) | 1.78 (1.03) | Group: F(2, 46)= 0.34, p=.72  **Phase: F_P_(1, 46)= 9.55, p=.003****  ACQ > EXT  GroupxPhase: F(2, 46)= 0.12, p=.89 |
|  | TC | [n=18] | - | 2.56 (1.12) | 2.08 (1.41) |  |
|  | HC | [n=15] | - | 2.63 (1.25) | 1.97 (1.34) |  |

**Supplementary Table 3b.** Mixed repeated measures ANOVAs (rmANOVA) across arousal ratings for each of the four conditions (ctx_unpred, ctx_safe, cue_pred, cue_safe) and each of the three phases (HAB, ACQ, EXT).

[**Abbreviations:** ACQ – Acquisition; CTX – Context; EXT – Extinction; HAB – Habituation; HC – Healthy control subjects without trauma experience; p_GG_ – Greenhouse-Geisser correction; pred – Predictable; PTSD – patients with PTSD; SCR – Skin conductance response; TC – healthy control subjects with trauma experience; unpred – Unpredictable]

| **Valence** | | | | | | |
| --- | --- | --- | --- | --- | --- | --- |
|  | **Groups** |  | **HAB** | **ACQ** | **EXT** | **Analyses** |
|  |  | **n** | **M (SD)** | **M (SD)** | **M (SD)** |  |
|  | **CTX_unpred** |  |  |  |  |  |
|  | PTSD | [n=14] | 3.54 (1.67) | 3.71 (1.99) | 3.36 (1.79) | Group: F(2, 41)= 0.30, p=.74  **Phase: F(2, 82)= 8.90, p_GG_<.001*****  ACQ > HAB + EXT  GroupxPhase: F(4, 82)= 0.92, p=.45 |
|  | TC | [n=17] | 3.26 (1.38) | 4.18 (2.08) | 3.18 (1.49) |  |
|  | HC | [n=13] | 2.81 (1.49) | 3.85 (1.63) | 2.81 (1.63) |  |
|  | **CTX_safe** | | | | | |
|  | PTSD | [n=16] | - | 3.53 (1.79) | 3.22 (1.81) | Group: F(2, 46)= 0.86, p=.43  Phase: F(1, 46)= 2.26, p=.14  **GroupxPhase: F(2, 46)= 3.69, p=.033***  HC_EXT_ > PTSD_EXT_ + TC_EXT_ |
|  | TC | [n=18] | - | 3.33 (1.37) | 3.72 (2.12) |  |
|  | HC | [n=15] | - | 3.40 (1.66) | 2.33 (0.96) |  |
|  | **CUE_pred** | | | | | |
|  | PTSD | [n=14] | 3.89 (1.91) | 3.63 (1.91) | 4.00 (2.12) | Group: F(2, 42)= 0.19, p=.83  Phase: F(2, 82)= 0.19, p=.83  GroupxPhase: F(4, 82)= 1.08, p=.37 |
|  | TC | [n=17] | 3.65 (1.43) | 3.65 (1.94) | 3.88 (2.18) |  |
|  | HC | [n=13] | 3.37 (1.91) | 3.81 (1.68) | 2.96 (1.89) |  |
|  | **CUE_safe** | | | | | |
|  | PTSD | [n=16] | - | 3.69 (1.71) | 3.84 (1.94) | Group: F(2, 46)= 1.83, p=.17  Phase: F_P_(1, 46)= 0.01, p=.94  GroupxPhase: F(2, 46)= 1.29, p=.29 |
|  | TC | [n=18] | - | 3.25 (1.31) | 3.61 (1.92) |  |
|  | HC | [n=15] | - | 3.00 (1.21) | 2.53 (1.70) |  |

**Supplementary Table 3c.** Mixed repeated measures ANOVAs (rmANOVA) across valence ratings for each of the four conditions (ctx_unpred, ctx_safe, cue_pred, cue_safe) and each of the three phases (HAB, ACQ, EXT).

[**Abbreviations:** ACQ – Acquisition; CTX – Context; EXT – Extinction; HAB – Habituation; HC – Healthy control subjects without trauma experience; p_GG_ – Greenhouse-Geisser correction; pred – Predictable; PTSD – patients with PTSD; SCR – Skin conductance response; TC – healthy control subjects with trauma experience; unpred – Unpredictable]

| **Contingency** | | | | | | |
| --- | --- | --- | --- | --- | --- | --- |
|  | **Groups** |  | **HAB** | **ACQ** | **EXT** | **Analyses** |
|  |  | **n** | **M (SD)** | **M (SD)** | **M (SD)** |  |
|  | **CTX_unpred** |  |  |  |  |  |
|  | PTSD | [n=14] | 2.64 (1.70) | 3.68 (1.87) | 2.11 (2.26) | Group: F(2, 41)= 1.12, p=.34  **Phase: F(2, 82)= 10.56, p_GG_<.001*****  EXT < HAB + ACQ  GroupxPhase: F(4, 82)= 0.55, p=.70 |
|  | TC | [n=17] | 3.12 (1.60) | 4.29 (1.98) | 2.38 (1.75) |  |
|  | HC | [n=13] | 3.08 (1.80) | 3.12 (2.58) | 1.58 (1.10) |  |
|  | **CTX_safe** | | | | | |
|  | PTSD | [n=16] | - | 3.22 (1.91) | 2.06 (2.15) | Group: F(2, 46)= 0.92, p=.41  **Phase: F(1, 46)= 13.36, p<.001*****  ACQ > EXT  GroupxPhase: F(2, 46)= 0.42, p=.66 |
|  | TC | [n=18] | - | 3.64 (1.75) | 2.64 (2.17) |  |
|  | HC | [n=15] | - | 3.33 (2.53) | 1.57 (0.89) |  |
|  | **CUE_pred** | | | | | |
|  | PTSD | [n=14] | 2.89 (1.96) | 2.75 (2.03) | 1.89 (1.71) | Group: F(2, 42)= 2.65, p=.83  **Phase: F(2, 82)= 5.27, p=.007****  EXT < HAB + ACQ  GroupxPhase: F(4, 82)= 0.53, p=.72 |
|  | TC | [n=17] | 3.41 (1.65) | 4.24 (2.22) | 2.79 (2.28) |  |
|  | HC | [n=13] | 2.81 (1.74) | 2.62 (1.96) | 1.85 (1.39) |  |
|  | **CUE_safe** | | | | | |
|  | PTSD | [n=16] | - | 2.78 (1.83) | 1.56 (1.14) | Group: F(2, 46)= 0.99, p=.38  **Phase: F_P_(1, 46)= 16.57, p<.001*****  ACQ > EXT  GroupxPhase: F(2, 46)= 0.29, p=.75 |
|  | TC | [n=18] | - | 2.94 (1.70) | 2.17 (1.56) |  |
|  | HC | [n=15] | - | 2.57 (1.46) | 1.43 (0.82) |  |

**Supplementary Table 3d.** Mixed repeated measures ANOVAs (rmANOVA) across contingency ratings for each of the four conditions (ctx_unpred, ctx_safe, cue_pred, cue_safe) and each of the three phases (HAB, ACQ, EXT).

[**Abbreviations:** ACQ – Acquisition; CTX – Context; EXT – Extinction; HAB – Habituation; HC – Healthy control subjects without trauma experience; p_GG_ – Greenhouse-Geisser correction; pred – Predictable; PTSD – patients with PTSD; SCR – Skin conductance response; TC – healthy control subjects with trauma experience; unpred – Unpredictable]

| **Ratings [Diff CS+-CS-]** | | | | | |
| --- | --- | --- | --- | --- | --- |
| **Arousal** | | **PTSD** | **TC** | **HC** | **Analyses** |
|  |  | [n=19] | [n=17] | [n=19] |  |
| HAB |  | 1.06 (1.53) | 0.95 (1.41) | 0.76 (1.51) | F(2, 52)= 0.18, p=.83 |
| ACQ | Con | 0.21 (1.87) | 0.20 (1.62) | -0.45 (1.40) | Group: F(2, 56)= 0.64, p=.53  **Phase: F(1, 56)= 39.13, p<.001*****  ACQ_cue_ > ACQ_con_  GroupxPhase: F(2, 56)= 0.15, p=.86 |
|  | Cue | 1.97 (1.97) | 1.75 (2.51) | 1.48 (2.42) |  |
| EXT | Con | 0.82 (1.67) | 0.15 (1.15) | 0.69 (1.62) | Group: F(2, 55)= 1.27, p=.29  Phase: F(1, 55)= 0.15, p=.70  GroupxPhase: F(2, 55)= 0.11, p=.90 |
|  | Cue | 0.88 (1.47) | 0.30 (1.41) | 0.67 (0.94) |  |
| **Valence** | | **PTSD** | **TC** | **HC** | **Analyses** |
|  |  | [n=19] | [n=17] | [n=19] |  |
| HAB |  | 0.85 (1.28) | 0.63 (1.63) | 0.26 (1.26) | F(2, 52)= 0.81, p=.45 |
| ACQ | Con | -0.13 (1.94) | 0.48 (1.60) | 0.48 (1.59) | Group: F(2, 56)= 1.11, p=.34  **Phase: F(1, 56)= 21.54, p<.001*****  ACQ_cue_ > ACQ_con_  GroupxPhase: F(2, 56)= 0.10, p=.90 |
|  | Cue | 1.71 (2.19) | 1.92 (2.12) | 1.25 (3.33) |  |
| EXT | Con | 0.35 (1.52) | 0.60 (1.26) | 0.79 (1.52) | Group: F(2, 55)= 0.23, p=.80  Phase: F(1, 55)= 0.26, p=.61  GroupxPhase: F(2, 55)= 1.98, p=.15 |
|  | Cue | 0.65 (1.61) | 0.95 (1.56) | 0.41 (1.06) |  |
| **Contingency** | | **PTSD** | **TC** | **HC** | **Analyses** |
|  |  | [n=20] | [n=19] | [n=20] |  |
| HAB |  | 1.18 (2.21) | 1.08 (1.73) | 0.82 (2.43) | F(2, 52)= 0.14, p=.87 |
| ACQ | Con | -0.45 (2.19) | 0.00 (2.73) | -0.42 (1.42 | Group: F(2, 56)= 0.37, p=.69  **Phase: F(1, 56)= 42.89, p<.001*****  ACQ_cue_ > ACQ_con_  GroupxPhase: F(2, 56)= 0.07, p=.93 |
|  | Cue | 2.89 (3.55) | 3.25 (3.38) | 2.50 (3.39) |  |
| EXT | Con | 0.68 (1.46) | 0.13 (1.44) | 0.57 (2.07) | Group: F(2, 55)= 0.70, p=.50  Phase: F(1, 55)= 0.37, p=.55  GroupxPhase: F(2, 55)= 1.04, p=.36 |
|  | Cue | 1.00 (2.24) | 0.53 (1.37) | 0.26 (0.96) |  |

**Suppl. Table 3e.** Difference of ratings between CS+ - CS- for the ratings of arousal**,** valence and contingency during all three phases (HAB, ACQ, EXT) and for all three groups (PTSD, TC, HC).

[**Abbreviations:** ACQ – Acquisition; Con – Context; CS – conditioned stimulus; EXT – Extinction; HAB – Habituation; HC – Healthy control subjects without trauma experience; PTSD – patients with PTSD; TC – healthy control subjects with trauma experience]
