# Supplementary material for: Altered frontolimbic activity during virtual reality-based contextual fear learning in patients with posttraumatic stress disorder
Source: Psychol Med. 2023 Jan 5;53(13):6345–55. doi: 10.1017/S0033291722003695 (PMC10520602; doi:10.1017/S0033291722003695)
Supplement: Supplementary file 1 [file S0033291722003695sup.zip › S0033291722003695sup008.docx]

| **SCR (in µS)** | |  |  |  |  |  |  | |  |
| --- | --- | --- | --- | --- | --- | --- | --- | --- | --- |
|  |  |  | **ctx**  **unpred** | **ctx**  **safe** |  | **Cue**  **pred** | | **Cue**  **safe** |  |
|  | **Groups** | **n** | **M (SD)** | **M (SD)** | **Analyses** | **M (SD)** | | **M (SD)** | **Analyses** |
|  | **ACQ** |  |  |  |  |  | |  |  |
|  | PTSD | [n=14] | 0.011  (0.014) | 0.006  (0.010) | Group: F(2, 36)= 3.24, p=.051  **Context: F(1, 36)= 14.55, p<.001*****  ctx_unpred > ctx_safe  Group x Context: F(2, 36)= 0.34, p=.71 | 0.028  (0.026) | | 0.007  (0.009) | **Group: F(2, 34)= 5.45, p=.009****  PTSD < HC + TC  **Context: F(1, 34)= 66.07, p<.001*****  cue_pred > cue_safe  Group x Context: F(2, 34)= 1.75, p=.19 |
|  | TC | [n=16] | 0.025  (0.022) | 0.017  (0.020) |  | 0.052  (0.028) | | 0.018  (0.015) |  |
|  | HC | [n=9] | 0.028  (0.019) | 0.020  (0.014) |  | 0.052  (0.009) | | 0.023  (0.015) |  |
|  | **EXT** |  |  |  |  |  | |  |  |
|  | PTSD | [n=12] | 0.012  (0.017) | 0.010  (0.014) | Group: F(2, 34)= 0.47, p=.63  Context: F(1, 34)= 2.47, p=.13  Group x Context: F(2, 35)= 0.25, p=.78 | 0.009  (0.013) | | 0.011  (0.014) | Group: F(2, 34)= 0.43, p=.65  Context: F(1, 34)= 1.31, p=.26  Group x Context: F(2, 34)= 1.67, p=.20 |
|  | TC | [n=16] | 0.015  (0.023) | 0.013  (0.024) |  | 0.015  (0.017) | | 0.013  (0.018) |  |
|  | HC | [n=9] | 0.024  (0.04) | 0.019  (0.027) |  | 0.017  (0.017) | | 0.014  (0.013) |  |

**Supplementary Table 4a.** Mixed repeated measures ANOVAs (rmANOVA) across SCRs for each of the two phases (context, cue) and each of the two phases (ACQ, EXT).

[**Abbreviations:** ACQ – Acquisition; CTX – Context; EXT – Extinction; HC – Healthy control subjects without trauma experience; pred – Predictable; PTSD – patients with PTSD; SCR – Skin conductance response; TC – healthy control subjects with trauma experience; unpred – Unpredictable; **µS** - Microsiemens]

| **SCR (in µS)**  **Diff. CS+-CS-** | |  |  |  |  |  |  | |  |
| --- | --- | --- | --- | --- | --- | --- | --- | --- | --- |
|  |  |  | **ctx**  **unpred** | **ctx**  **safe** |  | **Cue**  **pred** | | **Cue**  **pred** |  |
|  | **Groups** | **n** | **M (SD)** | **M (SD)** | **Analyses** | **M (SD)** | | **M (SD)** | **Analyses** |
|  | **ACQ** |  |  |  |  |  | |  |  |
|  | PTSD | [n=14] | 0.003  (0.010) | 0.000  (0.006) | **Group: F(2, 36)= 3.93, p=.029***  HC > PTSD + TC  Context: F(1, 36)= 0.83, p=.37  Group x Context: F(2, 36)= 0.36, p=.70 | 0.059  (0.062) | | 0.003  (0.007) | Group: F(2, 34)= 1.77, p=.19  **Context: F(1, 34)= 62.59, p<.001*****  cue_pred > cue_safe  Group x Context: F(2, 34)= 3.08, p=.059 |
|  | TC | [n=16] | 0.004  (0.013) | -0.002  (0.009) |  | 0.098  (0.046) | | 0.004  (0.026) |  |
|  | HC | [n=9] | 0.008  (0.015) | 0.009  (0.019) |  | 0.066  (0.029) | | 0.015  (0.023) |  |
|  | **EXT** |  |  |  |  |  | |  |  |
|  | PTSD | [n=12] | -0.017  (0.047) | 0.006  (0.016) | Group: F(2, 35)= 0.73, p=.49  **Context: F(1, 35)= 5.47, p=.025**  ctx_unpred_ext > ctx_safe_ext  Group x Context: F(2, 35)= 2.48, p=.098 | -0.001  (0.006) | | 0.000  (0.011) | Group: F(2, 34)= 0.87, p=.43  Context: F(1, 34)= 1.57, p=.22  Group x Context: F(2, 34)= 1.31, p=.28 |
|  | TC | [n=16] | 0.001  (0.001) | 0.002  (0.012) |  | 0.002  (0.009) | | 0.001  (0.015) |  |
|  | HC | [n=9] | -0.017  (0.047) | 0.006  (0.016) |  | -0.022  (0.076) | | 0.005  (0.014) |  |

**Supplementary Table 4b.** Mixed repeated measures ANOVAs (rmANOVA) across SCRs for each of the two phases (context, cue) and each of the two phases (ACQ, EXT).

[**Abbreviations:** ACQ – Acquisition; CTX – Context; EXT – Extinction; HC – Healthy control subjects without trauma experience; pred – Predictable; PTSD – patients with PTSD; SCR – Skin conductance response; TC – healthy control subjects with trauma experience; unpred – Unpredictable; **µS** - Microsiemens]
