# Supplementary material for: Altered frontolimbic activity during virtual reality-based contextual fear learning in patients with posttraumatic stress disorder
Source: Psychol Med. 2023 Jan 5;53(13):6345–55. doi: 10.1017/S0033291722003695 (PMC10520602; doi:10.1017/S0033291722003695)
Supplement: Supplementary file 1 [file S0033291722003695sup.zip › S0033291722003695sup009.docx]

| **Phase** | **Contexts** | **Contrast** | **k** | **Area of activation** | **MNI coordinates**  **(x,y,z)** | **p(cluster-level)** |
| --- | --- | --- | --- | --- | --- | --- |
| ACQ | ctx_unpred | HC>PTSD | 61 | Right middle cingulate gyrus | **9, -16, 29** | **.004*** |
|  |  |  | 43 | Left thalamus | -6, -7, 2 | .014 |
|  |  |  | 34 | Left parietal Operculum | -51, -25, 20 | .026 |
|  |  |  | 31 | Right supramarginal Gyrus | 48, -34, 32 | .032 |
|  | ctx_unpred > ctx_safectx | TC>PTSD | 37 | Right thalamus | 21, -19, 23 | .027 |
|  |  |  | 29 | Right caudate/accumbens | 6, 11, -4 | .046 |
| EXT | ctx_pred | TC>PTSD | 35 | Right anterior cingulate gyrus | 12, 41, 5 | .050 |
|  | ctx_unpred > ctx_safectx | PTSD>TC | 53 | Right caudate | 18, 23, 5 | .016 |
|  |  |  | 53 | Left caudate | -9, 20, 5 | .016 |
|  |  |  | 37 | Left hippocampus | -21, -40, 8 | .038 |

**Supplementary Table 5.** Whole brain contrasts for each of the two phases (ACQ, EXT) and between the three groups.

[**Abbreviations:** ACQ – Acquisition; CTX – Context; EXT – Extinction; HC – Healthy control subjects without trauma experience; MNI – coordinate system according to standard brains from the Montreal Neurological Institute; pred – Predictable; PTSD – patients with PTSD; TC – healthy control subjects with trauma experience; unpred – Unpredictable]

*cluster-level: p(FWE-corr.);
